# Abdominopelvic Mass Revealing Tuberculosis in a Young Woman

**DOI:** 10.1155/2021/7257533

**Published:** 2021-06-30

**Authors:** Hicham ELmajdoubi, Mariam EL Yahiyaoui, Marouane Baiss, Mohamed Bouzroud, El Mehdi Aboulfeth, Mohammed Najih, Sidi Mohamed Bouchentouf, Hakim ELkaoui, Ahmed Bounaim

**Affiliations:** ^1^Department of Digestive Surgery I, Mohammed V Military Hospital, 10100 Rabat, Morocco; ^2^Department of Pneumology, Moulay Youssef University Hospital Center, 11010 Rabat, Morocco

## Abstract

Tuberculosis is an infectious disease caused by Mycobacterium tuberculosis and remains a health problem, especially in developing countries. Abdominal location represents 5 to 10% of all locations. The clinical symptoms are not very specific, and the discovery of an abdominal mass in a context of deterioration of general state may wrongly lead to the diagnosis of a tumor. Radiological explorations remain sensitive in the detection of abdominal masses but they cannot prejudge their etiology. Surgical exploration is sometimes the only recourse either for diagnostic purposes or complications, and the diagnosis can only be confirmed by bacteriological and histological examinations of the surgical specimen. We report the case of abdominopelvic mass and lymph nodes revealing tuberculosis.

## 1. Introduction

Tuberculosis is a curable infectious disease but remains a health problem in developing countries. Its abdominal localization represents 5 to 10% of all locations [1].

Symptoms of abdominal pseudotumors are not specific, and diagnosis can often be missed and mimic malignancy. Tuberculosis must be evoked particularly in an environment with high endemicity.

Our observation concerns a case of pseudotumoral lymph node tuberculosis in a young woman confirmed by polymerase chain reaction (PCR) for detection of Mycobacterium tuberculosis and histological examination of the surgical specimen.

## 2. Case Report

**Figure 1:**
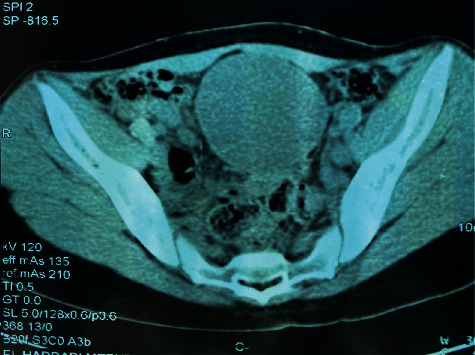
Abdominal computed tomography scan showing abdominopelvic mass.

**Figure 2:**
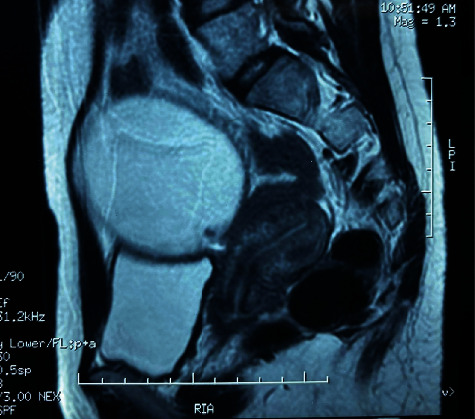
Magnetic resonance imaging showing abdominopelvic mass.

An 18-year-old female patient with no notable pathological history, consulted for chronic pain in the right iliac fossa evolving in a context of anorexia and weight loss of 10 kg in 4 months. On general examination, the patient was apyretic, conscious, and hemodynamically stable, and her conjunctiva was normally colored. The physical examination revealed a subumbilical mass mobile in both superficial and deep planes, undefined borders, and extending into the right iliac fossa; no hepatomegaly, splenomegaly, or lymphadenopathy was detected. Other systemic examinations were unremarkable. Her blood investigations were normal; her chest X-ray was also normal. The abdominal CT scan (Figure 1) showed two contiguous, well-limited, rounded formations with thick fluid content and thin, regular walls, they measured, respectively, 20 mm at the level of the right iliac fossa and 72 mm at the abdominopelvic level, under the umbilicus. The pelvic magnetic resonance imaging (Figure 2) showed a median intraperitoneal pelvic mass hyposignal on T1 and hypersignal on T2 and regular parietal enhancement after injection with mural nodules, it measured 77 × 81 × 80 mm; this mass was supravesical, coming into contact with the ileal intestines, the sigmoid, and the uterus behind it, keeping a separation line.

**Figure 3:**
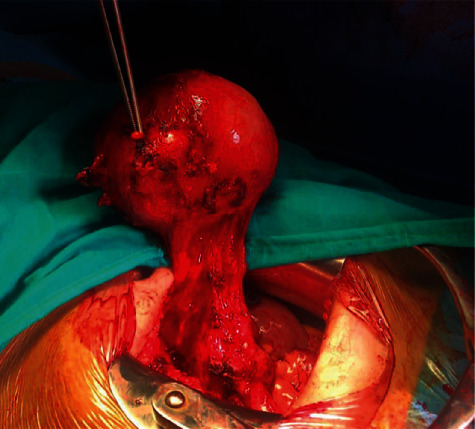
Intraoperative view of an abdominopelvic mass with satellite nodes.

**Figure 4:**
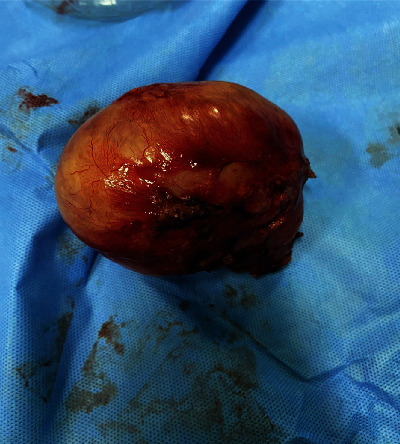
Operative specimen showing abdominopelvic mass.

Laparotomy exploration showed a mesenteric mass close to the ileocaecal region, solid hyper vascularized with some ileocaecal lymph nodes (Figure 3); the appendix and the ovaries appeared normal; a monobloc resection of the mass with the satellite lymph nodes was performed without intestinal resection (Figure 4). The postoperative course was simple, and she was discharged home after 4 days in good general condition.

**Figure 5:**
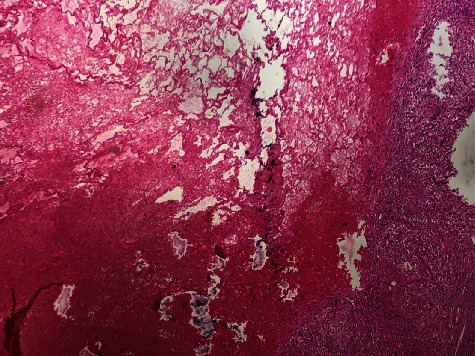
Microscopic pathologic examination showing epitheliogigantocellular granulomatous adenitis with a large liquefied necrosis in the center (HE, *G* ×50).

**Figure 6:**
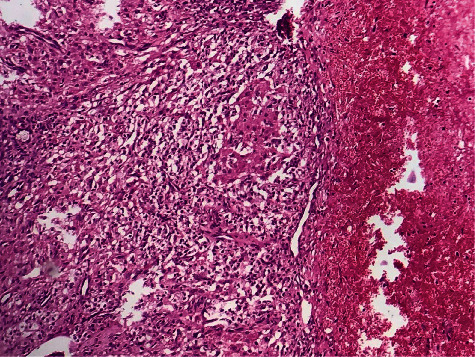
Granulomas in variable size (HE, *G* ×400).

Microscopic pathologic examination was in favor of a necrotizing granulomatous lymphadenitis which was highly indicative of tuberculosis (Figures 5 and 6). Polymerase chain reaction (PCR) test of the specimen was positive for Mycobacterium tuberculosis.

Quadruple therapy for tuberculosis with isoniazid, rifampicin, pyrazinamide, and ethambutol was prescribed for a two-month initial phase of treatment followed by a four-month continuation phase of isoniazid and rifampicin. In patient's follow-up, general condition was good and no problem was reported.

## 3. Discussion

Extrapulmonary tuberculosis represents an increasing percentage of all forms of tuberculosis, reaching 20-40% depending on the series [2], its abdominal location accounts for 5-10% of all locations, and abdominopelvic tuberculosis is the sixth most common form [1].

Tuberculosis lymph node involvement is rare in its primary abdominal localization, its encysted form remains an atypical presentation, and diagnosis can often be missed and mimic malignancy [3].

The diagnosis of this disease is difficult, and the symptomatology is very varied and not specific and may wrongly lead to tumoral pathology. The clinical features are dominated by the alteration of the general state (80%), abdominal pain (60%) and transit disorders (40%); the discovery of a palpable mass (20 to 25%) and more rarely a complication (occlusion, haemorrhage or perforation) may be revealing [4].

Chest X-ray shows suggestive images in case of associated pulmonary involvement [5].

On imaging studies, the diagnosis of pseudotumor tuberculosis is very difficult. The CT appearance of agglutinated bowel, parietal and surrounding organ infiltration, mesenteric adenopathy, and even peritoneal nodules is always suggestive of cancer in the first instance [6].

Abdominal CT is indeed sensitive in detecting abdominal masses but cannot prejudge their etiology.

The contribution of magnetic resonance imaging in this abdominal localization is nonspecific, this examination shows lesions in hyposignal T1 with a variable T2 signal during lymph node and visceral localizations [7]. These nonspecific radiological aspects can be seen in other pathologies; in the lymph node form, the diagnosis is made with lymphoma, lymph node metastases, or Whipple's disease. Digestive parietal involvement with agglutination of bowel may suggest a lymphoma, a carcinoid tumor, or even a carcinoma. In the solid organs, in particular the liver, the main differential diagnoses are represented by hepatocarcinoma in its hypodense form, lymphoma, hydatid cyst, and sarcoidosis [8].

Our patient presented a chronic abdominal pain in a context of deteriorating general condition, and the complementary examinations evoked in the first place an abdominopelvic cystic mass.

Surgery is sometimes the only recourse, either for diagnostic purposes or complications such as stenosis, occlusion, a compressive mass, perforation, or fistula. The surgical procedure depends on the operative finding and must remain as conservative as possible [9].

Surgical exploration will not determine the tubercular origin of these pseudotumoral forms, and diagnosis will only be made after bacteriological and histological examinations of the surgical specimen, as in the case of our patient [10].

Histopathological diagnosis of tuberculosis is usually observed in the presence of granulomatous inflammation and caseous necrosis. Molecular techniques such as PCR have high sensitivity and specificity for diagnosing tuberculosis [11].

Antituberculosis treatment should be initiated after bacteriological and anatomopathological confirmation on the surgical specimen for a period of six months: four drugs (isoniazide/rifampicin/pyrazinamide/ethambutol) for two months followed by two drugs (isoniazide/rifampicin) for four months, even for patients who are a priori cleared by surgical resection [12].

## 4. Conclusion

Abdominal pseudotumoral tuberculosis remains rare even in endemic areas, and its diagnosis is difficult because it can be confused on imaging with a tumor pathology, which often justifies recourse to surgical exploration, and the diagnosis is confirmed after bacteriological and histological examinations of the surgical specimen.

## References

[1] Rathi P., Gambhire P. (2016). Abdominal tuberculosis. *The Journal of the Association of Physicians of India*.

[2] Mazza-Stalder J., Nicod L., Janssens J. P. (2012). Extrapulmonary tuberculosis. *Revue des Maladies Respiratoires*.

[3] Fatemi S. R., Ghobakhlou M., Alizadeh L. (2014). Obstructive pseudotumor of tuberculosis in a young woman: a rare presentation. *Case Reports in Gastrointestinal Medicine*.

[4] El Barni R., Lahkim M., Achour A. (2012). La tuberculose abdominal pseudo-tumorale. *Pan Afr Med J*.

[5] Cagatay A. A., Caliskan Y., Aksoz S. (2004). Extrapulmonary tuberculosis in immunocompetent adults. *Scandinavian Journal of Infectious Diseases*.

[6] Vanhoenacker F. M., De Backer A. I., Op de Beeck B. (2004). Imaging of gastrointestinal and abdominal tuberculosis. *European Radiology*.

[7] da Rocha E. L., Pedrassa B. C., Bormann R. L., Kierszenbaum M. L., Torres L. R., D’Ippolito G. (2015). Abdominal tuberculosis: a radiological review with emphasis on computed tomography and magnetic resonance imaging findings. *Radiologia Brasileira*.

[8] Hablani N., Mhiri M. S., Graies K. T., Gharbi H. J., Abdallah S., Hamida R. B. H. (2005). Pseudotumoral form of abdominal tuberculosis: report of four cases. *Journal de Radiologie*.

[9] Fillion A., Ortega-Deballon P., Al-Samman S. (2016). Tuberculose abdominale dans une region de faible prevalence tuberculeuse. *Médecine et Maladies Infectieuses*.

[10] Ismaïli Z., Amraoui M., Mansouri F. (2006). Tuberculose colique pseudo-tumorale à double localisation. *Médecine du Maghreb*.

[11] Chawla K., Gupta S., Mukhopadhyay C., Rao P. S., Bhat S. S. (2009). PCR for M. tuberculosis in tissue samples. *The Journal of Infection in Developing Countries*.

[12] Evans R. P. T., Mourad M. M., Dvorkin L., Bramhall S. R. (2016). Hepatic and intra-abdominal tuberculosis: 2016 update. *Current Infectious Disease Reports*.

